# Nonallergic Asthma and Its Severity: Biomarkers for Its Discrimination in Peripheral Samples

**DOI:** 10.3389/fimmu.2018.01416

**Published:** 2018-06-21

**Authors:** Selene Baos, David Calzada, Lucía Cremades-Jimeno, Joaquín Sastre, César Picado, Joaquín Quiralte, Fernando Florido, Carlos Lahoz, Blanca Cárdaba

**Affiliations:** ^1^Immunology Department, Instituto de Investigación Sanitaria Fundación Jiménez Díaz, Universidad Autónoma de Madrid, Madrid, Spain; ^2^Centro de Investigación Biomédica en Red Enfermedades Respiratorias (CIBERES), Madrid, Spain; ^3^Allergy Department, Fundación Jiménez Díaz, Madrid, Spain; ^4^Service of Pneumology, Hospital Clinic, Universitat de Barcelona, Institut d’Investigacions Biomèdiques August Pi i Sunyer (IDIBAPS), Barcelona, Spain; ^5^Allergy Department, Hospital Universitario Virgen del Rocío, Seville, Spain; ^6^Allergy Department, Hospital Universitario San Cecilio, Granada, Spain

**Keywords:** biomarkers, gene expression, nonallergic asthma, protein expression, severity, receiver operating characteristic

## Abstract

Asthma is a complex and heterogeneous respiratory disorder characterized by chronic airway inflammation. It has generally been associated with allergic mechanisms related to type 2 airway inflammation. Nevertheless, between 10 and 33% of asthmatic individuals have nonallergic asthma (NA). Several targeted treatments are in clinical development for patients with Th2 immune response, but few biomarkers are been defined for low or non-Th2-mediated inflammation asthma. We have recently defined by gene expression a set of genes as potential biomarkers of NA, mainly associated with disease severity: IL10, MSR1, PHLDA1, SERPINB2, CHI3L1, IL8, and PI3. Here, we analyzed their protein expression and specificity using sera and isolated peripheral blood mononuclear cells (PBMCs). First, protein quantification was carried out using ELISA (in sera) or Western blot (proteins extracted from PBMCs by Trizol procedure), depending on the biomarker in 30 healthy controls (C) subjects and 30 NA patients. A receiver operating characteristic curve analysis was performed by using the R program to study the specificity and sensitivity of the candidate biomarkers at a gene- and protein expression level. Four kinds of comparisons were performed: total NA group vs C group, severe NA patients vs C, moderate–mild NA patients vs C, and severe NA patients vs moderate–mild NA patients. We found that all the single genes showed good sensitivity vs specificity for some phenotypic discrimination, with CHI3L1 and PI3 exhibiting the best results for C vs NA: CHI3L1 area under the curve (AUC) (CI 95%): 0.95 (0.84–1.00) and PI3 AUC: 0.99 (0.98–1.00); C vs severe NA: PI3 AUC: 1 (0.99–1.00); and C vs moderate–mild NA: CHI3L1 AUC: 1 (0.99–1.00) and PI3 AUC: 0.99 (0.96–1.00). However, the results for discriminating asthma disease and severity with protein expression were better when two or three biomarkers were combined. In conclusion, individual genes and combinations of proteins have been evaluated as reliable biomarkers for classifying NA subjects and their severity. These new panels could be good diagnostic tests.

## Introduction

Asthma is a complex respiratory disorder defined in the most recent Global Initiative for Asthma (1) as “a heterogeneous disease, usually characterized by chronic airway inflammation.” Asthma diagnosis is based on a history of respiratory symptoms, such as wheezing, shortness of breath, chest tightness, and cough, which varies over time and fluctuates in intensity. The World Health Organisation defines asthma as the most common chronic disease in children and estimates that more than 300 million people are affected (2). However, one of the major problems in defining this pathology is its wide clinical spectrum. It is generally accepted that clinical differences in treatment response and disease course are related to multiple underlying variations in genetic, pharmacologic, physiologic, biologic, and/or immunologic mechanisms that produce subclasses of phenotypes termed endotypes (3).

Despite this clinical heterogeneity, allergic mechanisms have been implicated in 50–80% of asthmatic patients and in approximately 50% of severe asthma (4, 5). Thus, asthma has generally been associated with type 2 airway inflammation characterized by elevated levels of immunoglobulin E, eosinophils, and several interleukins (IL), such as IL-4, IL-5, IL-13, and IL-9. Nevertheless, 10–33% of asthmatic individuals have nonallergic asthma (NA), or allergic sensitization that cannot be demonstrated (6). The generally accepted definition of NA includes negative skin prick or *in vitro*-specific IgE tests to a panel of local allergens, and at a minimum, a panel of perennial allergens; total serum IgE levels are typically normal or low (<150 IU/ml) (7). The mechanisms contributing to the non-type 2 immune response in asthmatic patients are less clear. Two major mechanisms leading to neutrophilic inflammation are postulated: dysregulated neutrophil-mediated immune responses due to respiratory infections (8) or defects in resolution of inflammation (9), and the activation of the IL-17-dependent pathway (10–14).

New strategies for the discovery and validation of biomarkers such as *omics* have been used to reveal the mechanisms responsible for asthma endotypes in different tissues. A biomarker is an objective, quantifiable biological parameter which serves as an index for health and physiological assessment. It could be the sign of a complex underlying *via* or an essential molecule associated directly with a main role in one endotype of a disease. Along these lines, many biomarkers targeted treatments are in clinical development for patients with Th2 immune response: anti-IL-4/IL-13, anti-IL-4, anti-IL-5, anti-IgE antibodies, and CRTh2 (chemoattractant receptor-homologous molecule expressed on Th2 cell antagonists) (15–17), although in the latter case, two recently conducted large Phase 2 studies with CRTh2 antagonists have either failed to demonstrate significant efficacy in clinical endpoints compared to placebo (18) or showed a similar degree of improvement with the active control (19) in patients with atopic/allergic asthma.

Besides, to date, no endotype-driven interventions have been proven effective for non-type 2 immune response asthma (20). In summary, more information is needed to optimize the patient’s therapeutic responses while avoiding adverse effects (20).

Against this backdrop, our research team has recently defined a group of genes that was differentially expressed in peripheral samples from nonallergic asthmatic patients (low or non-Th2 inflammation) and mainly associated with disease severity (21, 22). The current study assessing gene and protein biomarkers is a follow up of our previous study (21, 22). Here, we explore the relevance of the gene and protein expression of these potential biomarkers according to sensitivity and specificity analysis [receiver operating characteristic (ROC) curves]. The ultimate aim is to provide useful new biomarkers for the NA disease.

## Materials and Methods

### Subjects

The study population comprised 60 unrelated subjects: 30 healthy control subjects (C) and 30 patients with NA. The samples of the asthma group came from the asthma biobank of the CIBERES located at the IIS-Fundación Jiménez Díaz-UAM in Madrid (IIS-FJD-UAM) (21, 22). These patients were diagnosed with severe, moderate, or mild asthma according to the Spanish Guidelines for the Management of Asthma, or GEMA (23). The daily mean of inhaled corticosteroids during the last 6 months, previous the diagnosis, and while taking the sample was: 1,488 ± 541 µg in severe asthma, 1,100 ± 977.75 µg in moderate asthma, and 450 ± 463.68 µg in mild asthma. On the day of extraction of the sample, the subjects did not take any medication.

Pulmonary function tests were carried out by determining the predicted percentage of forced vital capacity (% FVC), forced expiratory volume in 1 s (% FEV_1_), and the post bronchodilator test (% PBD) or reversibility test.

The control subjects were healthy, with no history of respiratory diseases. They were diagnosed at the Allergy Service of two hospitals in Andalusia (Spain), *Vírgen del Rocío* University Hospital in Seville and *San Cecilio* University Hospital in Granada, and the samples were sent to the IIS-FJD-UAM to be processed.

All the subjects, controls, and NA patients, were tested by skin prick test against a panel of common allergens, including mites (*Dermatophagoides pteronyssinus, Dermatophagoides farinae*, and *Lepidoglyphus destructor*), epithelia (cat and dog), cockroaches (*Blatella orientalis* and *Blatella germanica*), pollens (*Cypress, banana shadow*, olive, mixture of grasses, *Artemisia, Parietaria*, and *Salsola*), and fungi (*Alternaria, Cladosporium, Aspergillus*, and *Penicillium*).

Informed consent in accordance with the Declaration of Helsinki was obtained from each subject. Ethical approval for the study was obtained from the ethical and research committees of the participating hospitals.

### Peripheral Blood Mononuclear Cells (PBMC) Isolation and Protein Extraction

Peripheral blood mononuclear cells were isolated from heparin-containing peripheral blood samples by gradient centrifugation using Lymphoprep (Comercial Rafer, Zaragoza, Spain) following the manufacturer’s instructions. PBMCs were isolated in steril conditions using endotoxin-free reagents. Total proteins were isolated from PBMCs (10^6^ cells) using the Trizol method (Invitrogen, Carlsbad, CA, USA). Protein levels were quantified by the BCA method (Thermo Fisher Scientific, Rockford, IL, USA).

### Gene Selection

*CD86, IL10, MSR1, PHLDA1, SERPINB2, CHI3L1, CPA3, IL8*, and *PI3* were selected as candidate biomarkers of the NA group (significance established at a relative gene quantification higher than 4 or lower than 0.25 comparing the C group) (21).

CD86 and CPA3 did not meet the strict criteria (RQ>4 or <0.25) in all of the comparisons (21), so we did not examine their expression at the protein level.

### Soluble Protein Level Analysis of IL-10, CHI3L1, IL-8, PI3, and POSTN

Soluble biomarkers with an ELISA commercial available were quantified through this technique.

Levels of IL-10, CHI3L1, IL-8, PI3, and POSTN were measured in the subjects’ serum using the human ELISA kits manufactured by ImmunoTools (Friesoythe, Germany) for IL-10; by R&D Systems (Minneapolis, MN, USA) for CHI3L1, PI3, and POSTN; and by Diaclone (Besancon Cedex, France) for IL-8. The procedure was carried out in accordance with each manufacturer’s protocol.

*POSTN* or periostin was analyzed at protein level given its relevance in the literature as a protein associated with asthma (24, 25).

### Protein Analysis of MSR1, PHLDA1, and SERPINB2

Protein determination of MSR1, PHLDA1, and SERPINB2 was performed by Western blot because they were not soluble proteins or no ELISA commercial kit was available for their study. MSR1 was analyzed in 9 C and 18 NA patients (8 severe NA patients and 10 with moderate–mild diagnosis), PHLDA1 was studied in 8 C and 5 NA (3 severe and 2 moderate–mild patients), and we studied SERPINB2 in 6 C and 11 NA subjects (6 with severe asthma and 5 with moderate–mild diagnosis). The Western blot procedure used was that of the Invitrogen Western Breeze^®^ Chemiluminescent Western Blot Immunodetection Kit (Life Technologies, Carlsbad, CA, USA) previously described (21). The primary antibody used to detect MSR1 was the rabbit anti-human polyclonal CD204/macrophage scavenger receptor I antibody (dilution 1:2,500) by ThermoFisher Scientific. PHLDA1 was detected with a rabbit anti-human polyclonal PHLDA1 antibody (Thermo Fisher Scientific) at a 1:500 dilution and SERPINB2 with the rabbit anti-human polyclonal SERPINB2 antibody by R&D Systems at a dilution of 1:250. Data of specific protein results were relative to β-Actin (dilution 1:1,000; Cell Signaling Techonology, Danvers, MA, USA) expression using the ImageQuant LAS 4000 (GE Healthcare Life Science).

### ROC Curve Analysis at the Gene and Protein Level

The ROC curve plots sensitivity vs specificity and the area under the curve (AUC) is an effective measure of accuracy for evaluating the diagnostic ability of tests to discriminate the true state of subjects, finding optimal cutoff values. A ROC curve was performed for the candidate biomarkers of the NA group, examining severity and expression at the genetic and protein level. Four kinds of comparisons were performed: total NA group vs C group, severe NA patients vs C group, moderate–mild NA patients vs C group, and severe NA patients vs moderate–mild NA patients. As a guide for interpreting the ROC curves, the following intervals have been established for AUC values: 0.50–0.60, poor test; 0.61–0.75, regular test; 0.76–0.90, good test; 0.91–0.97, very good test; 0.98–1, excellent test. Besides, only the results with a CI 95% between 0.70 and 1 were considered statistically relevant.

### Statistical Analysis

The levels and relative expression of the proteins studied were compared among groups by unpaired *t*-test, using the Graph-Pad InStat 3 program. Statistical significance was established in two-tailed *P* value <0.05. The ROC curve analyses were performed by using the R program.

## Results

### Subjects

The demographic and clinical parameters of the population studied are summarized in Table 1. The NA patients were significantly older than the C subjects. The mean levels of total IgE were similar between the two groups, 75.02 ± 128.21 IU/ml in healthy control subjects vs 82.04 ± 80.63 IU/ml in NA patients (*p*>0.05). However, % FEV_1_ and % FVC showed statistically significant differences between severe NA vs moderate–mild NA patients (66.33 ± 16.62 vs 85.38 ± 21.03, *P* = 0.0127 in % FEV_1_; 69.93 ± 19.94 vs 94 ± 19.52, *P* = 0.0031 in % FVC) (data not shown). Percentage and number of eosinophils in the NA group were normal (3.83 ± 2.24 vs 273.86 ± 137.13 cells/μl) (cutoff: 1–4% and 50–450 cells/μl). No significant differences were found in the presence of eosinophils between severe and moderate–mild NA subjects (percentage: 3.73 ± 2.48 vs 4.22 ± 1.25%, respectively; number: 264 ± 152.96 vs 310 ± 52.57 cells/μl, respectively) (data not shown). Skin prick test against a panel of common allergens was negative in all the participants in this study. Concomitant diseases in the NA group were: nonatopic rhinitis (80%), sinusitis (56%), polyposis (33.3%), and esophageal reflux (20%).

**Table 1:** Characteristics of the study population.

	*N*	Sex	Age	Smoking	Clinical diagnosis		Total IgE (IU/ml)	% FVC	% FVE_1_	% eosinophils	Number of eosinophils (cells/μl)
Control (C) subjects	30	67.86% women 32.14% men	45.66 ± 12.39	92.86% non-smokers 7.14% smokers 0% ex-smokers	100% healthy		75.02 ± 128.21	–	–	–	–

Nonallergic asthmatic (NA) subjects	30	73.33% women 26.67% men	58.03 ± 13.14^a^	73.33% non-smokers 10% smokers 16.67% ex-smokers	50% severe asthma 30% moderate asthma 20% mild asthma	No allergic symptoms	82.04 ± 80.63	81.11 ± 22.69	75.18 ± 22.82	3.83 ± 2.24	273.86 ± 137.13

*% FVC: percentage of predicted value of forced vital capacity; % FEV_1_: percentage of predicted value of forced expiratory volume in 1 s*.

*^a^Statistically significant comparison (*P* < 0.05) between C and NA group*.

### ROC Curve Analysis at the Genetic Level

The genes studied were grouped into five categories based on the ROC curve analysis results (Table 2) (see Material and Methods).

**Table 2:** Classification of biomarkers by the receiver operating characteristic (ROC) curves analysis.

Comparison/AUC (CI 95%)	Excellent test (0.98–1)	Very good (0.91–0.97)	Good (0.76–0.90)	Regular (0.61–0.75)	Poor (0.5–0.60)

(A) Gene expression level					
C vs NA	PI3, 0.99	CHI3L1, 0.95	IL8, 0.90 MSR1, 0.89 CPA3, 0.88 IL10, 0.87 SERPINB2, 0.84 PHDLA1, 0.83 CD86, 0.81		

C vs MM	CHI3L1, 1 PI3, 0.99	IL8, 0.91	CPA3, 0.88 SERPINB2, 0.86 MSR1, 0.82 IL10, 0.79 PHDLA1, 0.77	CD86, 0.71	

C vs S	PI3, 1	MSR1, 0.94 IL10, 0.94 CD86, 0.91	CHI3L1, 0.89 IL8, 0.89 PHLDA1, 0.89 CPA3, 0.88 SERPINB2, 0.82		

MM vs S			CD86, 0.78	MSR1, 0.66 CPA3, 0.65 PHDLA1, 0.62	PI3, 0.60 IL8, 0.53 IL 10, 0.51 SERPINB2, 0.51

**(B) Protein expression level**					

C vs NA		MSR1 low, 0.96 SERPINB2, 0.91		POSTN, 0.62	CHI3L1, 0.6 MSR1 up, 0.6 IL8, 0.5 PI3, 0.5 IL10, PHLDA1<0.5

C vs S		MSR1 low, 0.93 SERPINB2, 0.93		CHI3L1, 0.63 IL8, 0.62	MSR1 up, 0.60 IL10, 0.56 POSTN, 0.55 PI3, 0.52 PHLDA1, ND

C vs MM	MSR1 low, 1		SERPINB2, 0.89	POSTN, 0.69 IL8, 0.62	MSR1 up, 0.6 CHI3L1, 0.56 PI3, 0.51 IL10<0.5 PHLDA1, ND

MM vs S			IL8, 0.76	SERPINB2, 0.7 POSTN, 0.65 CHI3L1, 0.62	PI3, 0.55 MSR1 low, 0.55 IL10, MSR1 up<0.5 PHLDA1, ND

*Summary of the ROC curve analysis of the most important biomarkers by comparison. AUC: area under the curve. ND: non-determined because of lack of data. C: control group. NA: total nonallergic asthma group. MM: group of subjects with moderate–mild asthma. S: group of subjects with severe asthma. MSR1 low: MSR1’s lower band protein expression. MSR1 up: MSR1’s upper band protein expression*.

Comparing the total subjects of the C and NA groups, we found that all genes fell into the “good” test category, except *CHI3L1* and *PI3* which obtained very good and excellent ratings, respectively. The results according to the severity of the NA group varied. When comparing C to moderate/mild NA subjects, *MSR1, IL10, CPA3, PHLDA1*, and *SERPINB2* maintained their “good” status, while *IL8* moved to a better ranking (very good), and *CD86* lowered to “regular.” *PI3* also maintained its excellent test position, joined by *CHI3L1*. The comparisons between C and severe NA patients differed from the overall analysis in some of the genes studied. While *IL8, CPA3, PHLDA1*, and *SERPINB2* stayed in the good test category and *PI3* continued to be excellent, *MSR1, IL10*, and *CD86* moved up to the “very good” test position, and *CHI3L1* was lowered to “good.”

*CD86* was the only good biomarker for asthma-severity discrimination. The rest of the genes were found to be “regular” or “poor” for discriminating moderate–mild NA from severe NA patients.

### Protein Expression

**Figure 1:**
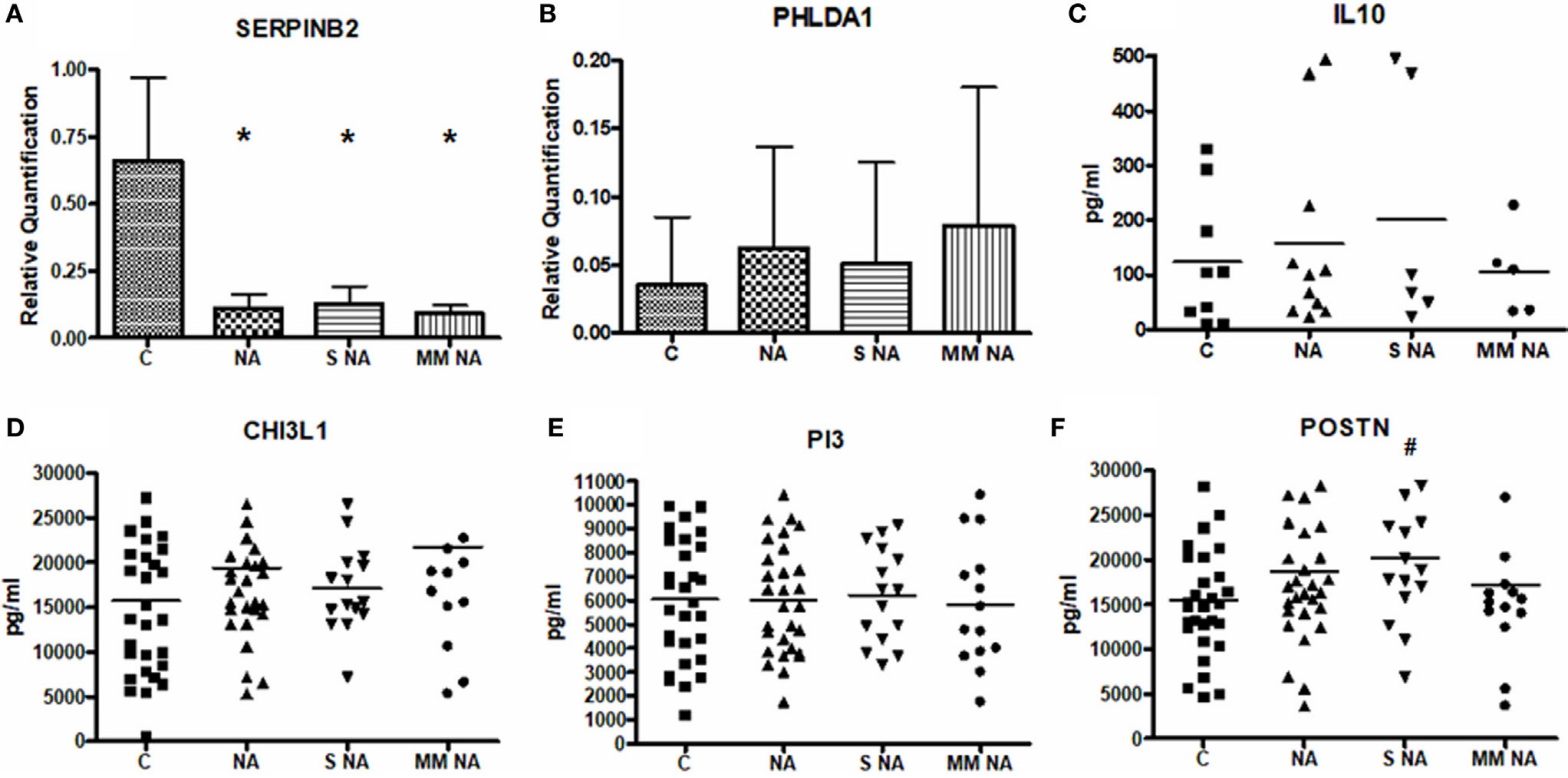
Mean levels of the protein expression. **(A)** Mean levels of SERPINB2. **(B)** Mean levels of PHLDA1. **(C)** Mean levels of IL10. **(D)** Mean levels of CHI3L1. **(E)** Mean levels of PI3. **(F)** Mean levels of POSTN. *Statistically significant comparison (*P* < 0.0001) between the C and the group selected. #Statistically significant comparison (*P* < 0.05) between the C and the group selected. Protein levels of SERPINB2 and PHLDA1 were measured by Western Blot in 6 C and 11 NA subjects (6 with severe asthma and 5 with moderate–mild diagnosis) and 8 C and 5 NA (3 severe and 2 moderate–mild patients), respectively. Densitometric analysis was done in individual blots (by the ImageQuant LAS 4000 software, as it is explained in Section “Materials and Methods”) using β-actin protein for normalization. IL-10, CHI3L1, PI3, and POSTN were quantified by ELISA in all the patients of the study population. Abbreviations: C, control group; NA, total nonallergic asthma group; S NA, group of subjects with severe asthma; MM NA, group of subjects with moderate–mild asthma.

SERPINB2 and PHLDA1 were quantified in the total protein extracted from PBMCs. Levels of IL-10, CHI3L1, PI3, and POSTN proteins were measured in the sera of the study population. Relative quantification and mean sera levels (expressed as pg/ml) are summarized in Figure 1. Only SERPINB2 and POSTN showed statistically significant differences. For SERPINB2, the control group (0.66 ± 0.31) had a higher expression than the total NA patients (0.11 ± 0.05, *P* < 0.0001). These differences were also shown when the C group was compared with the severe NA (0.13 ± 0.07, *P* = 0.0019) and moderate–mild NA (0.09 ± 0.03, *P* = 0.0029) groups. In contrast, the protein levels of POSTN were higher in the total NA group (18,679.59 ± 8,086.07 pg/ml), as well as in the severe (20,198.91 ± 7,859.24 pg/ml) and, in the moderate–mild NA patients (17,160.28 ± 7,930.59 pg/ml) compared to the C group (15,487.71 ± 6,532.85 pg/ml), but only in the severe asthma patients were the differences statistically significant.

Though there were no statistically significant differences in the other proteins studied, a tendency was observed when the NA patients were analyzed according to severity. The IL-10 levels were higher in severe (200.60 ± 219.85 pg/ml) diagnosed subjects compared to C subjects (123.38 ± 120.61 pg/ml) and to moderate–mild NA patients (105.27 ± 79.62 pg/ml). This same tendency was observed in PI3: severe NA group: 6,225.03 ± 1,999.11 pg/ml, moderate–mild NA group: 5,824.14 ± 2,624.29 pg/ml, and C subjects: 6,056.30 ± 2,535.83 pg/ml. In contrast, PHLDA1 and CHI3L1 showed the highest levels in moderate–mild NA patients (moderate–mild NA: 0.08 ± 0.10; severe NA: 0.05 ± 0.07; C: 0.04 ± 0.05 for PHLDA1; and moderate–mild NA: 21,702.56 ± 11,589.97 pg/ml; severe NA: 17,026.99 ± 4,845.20 pg/ml; C: 15,729.18 ± 8,576.85 pg/ml for CHI3L1). Protein expression of MSR1 and IL-8 were previously discussed (22). Briefly, IL-8 protein expression showed slight but non-significant differences between severe (505.49 ± 387.8 pg/ml) and moderate–mild (377.7 ± 338.27 pg/ml) NA patients; and for MSR1 two proteic bands were found by Western blot, with distinct behaviors. The lower molecular weight band showed statistical differences between C subjects and NA subjects.

### ROC Curve Analysis at the Protein Level

The MSR1’s lower band and SERPINB2 were the best individual biomarkers at the protein level according to the ROC curve results (Table 2). These two biomarkers were classified as “very good” for comparing the whole group of NA and severe patients vs the control group. For discriminating the moderate–mild patients from controls, the MSR1’s lower band was “excellent” and SERPINB2 was “good.”

Overall, all other biomarkers fell into the “poor” or “regular” test category (Table 2). PI3, IL-10, and MSR1’s upper band would be considered poor biomarkers. The same was the case for CHI3L1 and IL-8, except when classifying severe NA patients and when discriminating moderate–mild from severe patients in the case of CHI3L1, and for IL-8 (when comparing moderate–mild and severe patients to healthy subjects), whose expression rose to “regular.” Additionally, IL-8 was able to discriminate moderate–mild from severe patients with a good AUC value (0.76). POSTN was classified as “regular” when comparing the total and the moderate–mild NA subjects and “poor” for the severe patients.

Given the poor results obtained in the individual ROC curve study of several biomarkers, an analysis of the AUC values combining two and three biomarkers was carried out.

The results of the analysis combining two proteins are summarized in Table 3. There was an improvement of the sensitivity and the specificity or synergy when several biomarkers were combined in the discrimination of total NA vs C groups (Table 3A): this is the case for MSR1’s upper band + MSR1’s lower band, CHI3L1 + MSR1’s upper band and POSTN + PHLDA1.

**Table 3:** Receiver operating characteristic (ROC) curve analyses of the protein expression combining two biomarkers.

	CHI3L1	IL-10	IL-8	PI3	POSTN	MSR1 up	MSR1 low	PHLDA1	SERPINB2

(A) Total nonallergic asthma (NA) group compared with the control group									
CHI3L1	0.60	0.48	0.67	0.59	0.63	**0.78**	**0.97**	0.70	**0.92**
IL-10	–	0.42	ND	0.59	0.47	ND	ND	ND	ND
IL-8	–	ND	0.50	0.61	0.74	ND	ND	ND	ND
PI3	–	–	–	0.50	0.67	0.63	**0.96**	0.47	**0.91**
POSTN	–	–	–	–	0.62	0.73	**0.96**	**0.82**	**0.95**
MSR1 up	–	ND	ND	–	–	0.60	**1**	ND	ND
MSR1 low	–	ND	ND	–	–	–	**0.96**	ND	ND
PHLDA1	–	ND	ND	–	–	ND	ND	0.42	ND
SERPINB2	–	ND	ND	–	–	ND	ND	ND	**0.91**

**(B) Moderate–mild NA group compared with the control group**									

CHI3L1	0.56	0.41	0.63	0.56	0.70	**0.83**	ND	ND	**0.97**
IL-10	–	0.41	ND	0.67	0.63	ND	ND	ND	ND
IL-8	–	ND	0.62	**0.76**	**0.87**	ND	ND	ND	ND
PI3	–	–	–	0.51	0.69	0.61	ND	ND	**0.89**
POSTN	–	–	–	–	0.69	**0.81**	ND	ND	**0.97**
MSR1 up	–	ND	ND	–	–	0.60	ND	ND	ND
MSR1 low	ND	ND	ND	ND	ND	ND	**1**	ND	ND
PHLDA1	ND	ND	ND	ND	ND	ND	ND	0.48	ND
SERPINB2	–	ND	ND	–	–	ND	ND	ND	**0.89**

**(C) Severe NA group compared with the control group**									

CHI3L1	0.63	0.62	0.67	0.63	0.64	**0.76**	**0.94**	ND	**0.93**
IL-10	–	0.56	ND	0.51	0.60	ND	ND	ND	ND
IL-8	–	ND	0.62	0.46	0.57	ND	ND	ND	ND
PI3	–	–	–	0.52	0.65	0.64	**0.95**	ND	**0.93**
POSTN	–	–	–	–	0.55	0.66	**0.93**	ND	**0.97**
MSR1 up	–	ND	ND	–	–	0.60	**1**	ND	ND
MSR1 low	–	ND	ND	–	–	–	**0.93**	ND	ND
PHLDA1	ND	ND	ND	ND	ND	ND	ND	0.48	ND
SERPINB2	–	ND	ND	–	–	ND	ND	ND	**0.93**

**(D) Moderate–mild NA group compared with the severe group**									

CHI3L1	0.62	0.43	**0.76**	0.56	**0.77**	0.68	0.65	ND	0.63
IL-10	–	0.47	ND	0.63	**0.77**	ND	ND	ND	ND
IL-8	–	ND	**0.76**	**0.82**	**0.78**	ND	ND	ND	ND
PI3	–	–	–	0.55	0.57	0.60	0.60	ND	0.73
POSTN	–	–	–	–	0.65	0.64	0.62	ND	**0.90**
MSR1 up	–	ND	ND	–	–	0.46	0.55	ND	ND
MSR1 low	–	ND	ND	–	–	–	0.55	ND	ND
PHLDA1	ND	ND	ND	ND	ND	ND	ND	0.48	ND
SERPINB2	–	ND	ND	–	–	ND	ND	ND	0.70

*In bold are the area under the curve values greater than 0.75, which are considered good, very good, or excellent tests. ND: non-determined because of lack of data. Cells with a dash refer to a comparison already shown in another cell of this table. MSR1 low: MSR1’s lower band protein expression. MSR1 up: MSR1’s upper band protein expression. In gray are indicated the individual ROC curve analysis of the protein expression of the nine biomarkers*.

When the analyses performed were comparing moderate–mild NA subjects and the C group (Table 3B), the results showed strong synergies between CHI3L1 + MSR1’s upper band, CHI3L1 + SERPINB2, IL-8 + PI3, IL-8 + POSTN, POSTN + MSR1’s upper band, and POSTN + SERPINB2. In the comparison of severe NA subjects (Table 3C) with C group, the improvement of the AUC values was observed in the MSR1’s upper band + MSR1’s lower band, CHI3L1 + MSR1’s lower band, and in POSTN + SERPINB2.

The combination of three biomarkers (Table 4) gave very interesting combinations, with AUC values over 0.75, meaning that good, very good, or excellent test were found for discriminating asthma and its severity from the control population. In the case of the total NA group (Table 4A), the following synergies were of great sensitivity and specificity: CHI3L1 + PI3 + MSR1’s upper band, CHI3L1 + POSTN + MSR1’s upper band, CHI3L1 + POSTN + PHLDA1, CHI3L1 + POSTN + SERPINB2, PI3 + POSTN + MSR1’s upper band, PI3 + POSTN + PHLDA1, and PI3 + POSTN + SERPINB2. The analysis of the moderate–mild NA patients compared with the C group (Table 4A) shared several combinations of biomarkers with the total NA group: CHI3L1 + PI3 + MSR1’s upper band, PI3 + POSTN + MSR1’s upper band, and PI3 + POSTN + SERPINB2, but particular of this comparison were CHI3L1 + IL-8 + PI3, CHI3L1 + IL-8 + POSTN, and IL-8 + PI3 + POSTN. The discrimination of severe patients from control subjects shared combinations of biomarkers with the other two comparisons. Important synergies were observed with PI3 + POSTN + MSR1’s upper band and PI3 + POSTN + MSR1’s lower band. As well as in the moderate–mild patients, CHI3L1 + IL-8 + PI3 was an interesting combination for discriminating severe subjects from C group.

**Table 4:** Receiver operating characteristic (ROC) curve analysis of the protein expression combining three biomarkers.

(A) NA compared with the C			

Combination of biomarkers	NA vs C area under the curve (AUC) value	MM vs C AUC value	S vs C AUC value
CHI3L1 + IL-10 + PI3	0.59	0.67	0.64
CHI3L1 + IL-10 + POSTN	0.53	0.63	0.64
CHI3L1 + IL-8 + PI3	0.67	**0.79**	**0.79**
CHI3L1 + IL-8 + POSTN	0.72	**0.87**	0.67
CHI3L1 + PI3 + POSTN	0.66	0.68	0.62
CHI3L1 + PI3 + MSR1 up	**0.77**	**0.83**	0.64
CHI3L1 + PI3 + MSR1 low	**0.97**	ND	**0.95**
CHI3L1 + PI3 + PHLDA1	0.70	ND	ND
CHI3L1 + PI3 + SERPINB2	**0.92**	ND	**0.93**
CHI3L1 + POSTN + MSR1 up	**0.83**	**0.88**	**0.76**
CHI3L1 + POSTN + MSR1 low	**0.97**	ND	**0.94**
CHI3L1 + POSTN + PHLDA1	**0.78**	ND	ND
CHI3L1 + POSTN + SERPINB2	**0.94**	ND	ND
IL-10 + PI3 + POSTN	0.57	0.61	0.60
IL-8 + PI3 + POSTN	0.75	**0.84**	0.62
PI3 + POSTN + MSR1 up	**0.77**	**0.81**	**0.77**
PI3 + POSTN + MSR1 low	**0.97**	ND	**0.96**
PI3 + POSTN + PHLDA1	**0.78**	ND	ND
PI3 + POSTN + SERPINB2	**0.94**	**0.97**	ND

**(B) Moderate–mild NA compared with the severe group**			

**Combination of biomarkers**			**AUC value**

CHI3L1 + IL-10 + PI3			0.63
CHI3L1 + IL-10 + POSTN			**0.77**
CHI3L1 + IL-8 + PI3			**0.88**
CHI3L1 + IL-8 + POSTN			**0.98**
CHI3L1 + PI3 + POSTN			**0.76**
CHI3L1 + PI3 + MSR1 up			**0.78**
CHI3L1 + PI3 + MSR1 low			0.68
CHI3L1 + PI3 + SERPINB2			0.73
CHI3L1 + POSTN + MSR1 up			**0.80**
CHI3L1 + POSTN + MSR1 low			**0.80**
CHI3L1 + MSR1 up + MSR1 low			0.68
IL-10 + PI3 + POSTN			**0.77**
IL-8 + PI3 + POSTN			**0.82**
PI3 + POSTN + MSR1 up			0.60
PI3 + POSTN + MSR1 low			0.60
PI3 + POSTN + SERPINB2			**0.93**
PI3 + MSR1 up + MSR1 low			0.60
POSTN + MSR1 up + MSR1 low			0.62

*In bold are the AUC values greater than 0.75, which are considered good, very good, or excellent tests. C: control group. NA: total nonallergic asthma group. MM: group of subjects with moderate–mild asthma. S: group of subjects with severe asthma. MSR1 low: MSR1’s lower band protein expression. MSR1 up: MSR1’s upper band protein expression*.

The comparison between the moderate–mild NA and severe NA subjects was also performed (Table 2, Table 3D, and Table 4B). In the individual analysis, all the biomarkers were classified as “regular” or “poor,” except IL-8, whose AUC value was good. When two biomarkers were combined, the combinations of CHI3L1 + POSTN, IL10 + POSTN, IL-8 + PI3, and SERPINB2 + POSTN were good to discriminate severe from moderate–mild asthma, and of smaller importance was the grouping of IL-8 + POSTN. In the three-biomarker analysis, the list of important synergies is longer (Table 4B).

There was an improvement of the sensitivity and the specificity or synergy when several biomarkers were combined, obtaining very interesting combinations, with AUC values over 0.75, meaning that good, very good, or excellent test were found for discriminating phenotypic conditions. Bringing together all the results, we established a ranking of the best biomarkers or cluster of biomarkers able to discriminate each condition analyzed, with a predictive accuracy of at least very good (AUC > 0.75). These rankings are shown in Table 5. It is worth highlighting the permanent presence of POSTN in most of the combinations able to discriminate moderate–mild and severe NA patients, as well as the best combination for this discrimination, CHI3L1 + IL-8 + POSTN.

**Table 5:** Ranking of the best individual and combined proteic biomarkers for each discrimination.

	Area under the curve (AUC) (IC 95%) value	Threshold
**(A) Biomarkers able to discriminate NA patients from C group**		
MSR1 low SERPINB2	**0.96 (0.89–1.00)** **0.91 (0.72–1.00)**	0.148 0.404
MSR1 low + MSR1 up CHI3L1 + MSR1 low POSTN + SERPINB2 CHI3L1 + SERPINB2 POSTN + PHLDA1 CHI3L1 + MSR1 up	**1** **0.97 (0.91–1.00)** **0.95 (0.85–1.00)** **0.92 (0.77–1.00)** 0.82 (0.59–1.00) 0.78 (0.60–0.95)	0.148, 0.208 13,064, 0.148 13,633, 0.404 13,064, 0.404 13,633, 0.011 13,064, 0.208
PI3 + POSTN + MSR1 low CHI3L1 + POSTN + MSR1 up PI3 + POSTN + MSR1 up	**0.97 (0.90–1.00)** 0.83 (0.67–0.98) 0.77 (0.58–0.96)	6,528, 13,633, 0.148 13,064, 13,633, 0.208 6,528, 13,633, 0.208

**(B) Biomarkers able to discriminate the moderate/mild NA patients from the C group**		

MSR1 low SERPINB2	**1** 0.89 (0.66–1.00)	0.148 0.404
CHI3L1 + SERPINB2 POSTN + SERPINB2 IL-8 + POSTN CHI3L1 + MSR1 up POSTN + MSR1 up IL-8 + PI3	**0.97 (0.90–1.00)** **0.97 (0.90–1.00)** **0.87 (0.70–1.00)** 0.83 (0.63–1.00) 0.81 (0.58–1.00) 0.76 (0.50–1.00)	13,064, 0.404 15,787, 0.404 677, 15,787 13,064, 0.208 15,787, 0.208 677, 3,074
CHI3L1 + IL-8 + POSTN CHI3L1 + IL-8 + PI3	**0.87 (0.70–1.00)** 0.79 (0.55–1.00)	13,064, 677, 15,787 13,064, 677, 3,074

**(C) Biomarkers able to discriminate the severe NA patients from the C group**		

MSR1 low SERPINB2	**0.93 (0.80–1.00)** **0.93 (0.78–1.00)**	0.137 0.359
MSR1 low + MSR1 up POSTN + SERPINB2 PI3 + MSR1 low CHI3L1 + MSR1 low CHI3L1 + MSR1 up	**1** **0.97 (0.87–1.00)** **0.95 (0.85–1.00)** **0.94 (0.83–1.00)** 0.76 (0.50–1.00)	0.137, 0.254 13,633, 0.359 6,528, 0.137 14,367, 0.137 14,367, 0.254
PI3 + POSTN + MSR1 low CHI3L1 + PI3 + MSR1 low CHI3L1 + IL-8 + PI3 PI3 + POSTN + MSR1 up	**0.96 (0.88–1.00)** **0.95 (0.86–1.00)** 0.79 (0.56–1.00) 0.77 (0.54–0.99)	6,528, 13,633, 0.137 14,367, 6,528, 0.137 14,367, 262, 6,528 6,528, 13,633, 0.254

**(D) Biomarkers able to discriminate the moderate/mild NA patients from the severe NA group**		

IL-8	0.76 (0.49–1.00)	841
POSTN + SERPINB2 IL-8 + PI3 IL-8 + POSTN CHI3L1 + POSTN IL-10 + POSTN	**0.90 (0.71–1.00)** 0.82 (0.58–1.00) 0.78 (0.52–1.00) 0.77 (0.59–0.96) 0.77 (0.43–1.00)	17,419, 0.132 841, 4,845 841, 17,419 18,500, 17,419 105.2, 17,419
CHI3L1 + IL-8 + POSTN PI3 + POSTN + SERPINB2 CHI3L1 + IL-8 + PI3 CHI3L1 + POSTN + MSR1 up CHI3L1 + POSTN + MSR1 low CHI3L1 + PI3 + MSR1 up	**0.98 (0.92–1.00)** **0.93 (0.78–1.00)** 0.88 (0.69–1.00) 0.80 (0.57–1.00) 0.80 (0.57–1.00) 0.78 (0.53–1.00)	18,500, 841, 17,419 4,845, 17,419, 0.132 18,500, 841, 4,845 18,500, 17,419, 0.257 18,500, 17,419, 0.056 18,500, 4,845, 0.257

*Summary of the best options for discriminating each condition, obtained from the ROC curve analysis of protein expression from each of the nine biomarkers, alone or in combination. C: control group. NA: nonallergic asthma group. MM: group of subjects with moderate–mild asthma. S: group of subjects with severe asthma. MSR1 low: MSR1’s lower band protein expression. MSR1 up: MSR1’s upper band protein expression. Threshold means the proteic levels of each biomarker able to discriminate each condition, with the AUC indicated. In bold are marked the options with the best statistical power (CI 95% between 0.7 and 1)*.

## Discussion

There is a real need to improve the diagnosis and treatment of the asthmatic disease. Many efforts are being undertaken to define new biological therapies against specific targets that define asthma mediated by Th2 inflammation; however, a substantial number of asthmatic patients present low or non-Th2 inflammation. We recently defined a group of genes differentially expressed in peripheral samples from nonallergic asthmatic patients (low or non-Th2 inflammation) and some of them, mainly associated with the severity of these diseases. In this report, we further explore the relevance of the gene and protein expression of these potential biomarkers, through the analysis of their individual and/or combined expression, in order to demonstrate their ability to discriminate asthma disease and severity. This study seeks to provide different panels of biomarkers associated with NA disease that could be useful for the diagnosis and/or therapy of this phenotype of asthma.

The ideal biomarker should be sensitive, specific, simple to perform, non-invasive, and inexpensive if possible (26). In the present report, we have evaluated the potential of nine genes and proteins to serve as biomarkers using peripheral blood samples from healthy controls and nonallergic asthmatic patients. We first analyzed their ability to discriminate asthmatic patients from healthy controls; second, we examined their potential as biomarkers of the degree of severity, comparing severe and/or moderate–mild patients with healthy control subjects; and last, we studied their potential to discriminate severity from moderate–mild in nonallergic asthmatic disease. As summarized in Table 2, all the genes were able to discriminate any of the phenotypical condition (NA, severe NA, or mild/moderate NA) with AUC values from excellent to good (ranging from 1 to 0.77), with the only exception of *CD86*, which was the poor biomarker for discriminating moderate/mild NA patients from controls (AUC: 0.71). According to these data, *PI3, CHI3L1*, and *IL8* are the best gene biomarkers (excellent or very good AUC) for discriminating NA from healthy control subjects, as well as for discriminating NA moderate/mild patients from control subjects. These results are in concordance with the recent description of the protective effect of *PI3* against adult asthma (27). *PI3* or Elafin, is a potent inhibitor of serine proteases, which plays a central role in controlling excessive activity of neutrophil elastase. It is a modulator of many parameters that are critical for inflammation, although it has pleiotropic effects (28). *CHI3L1* or YKL-40 is thought to play a role in tissue inflammation and remodeling (29), and its role as a possible biomarker has been reviewed in YKL-40 regulated signaling mechanisms (30). Also, correlations between YKL-40 levels and neutrophilic inflammation have been described (31). Finally, *IL8* (a member of the CXC chemokines) is considered to be one of the main mediators of the inflammatory response and very important for the survival and chemotaxis of neutrophils. It is secreted by several cell types and has been associated with several respiratory disorders (32).

These three biomarkers (*PI3, CHI3L1*, and *IL8*) are closely related to neutrophils, suggesting the relevance of this kind of cells in noneosinophilic nonallergic asthmatic disease and severity (data here cannot be demonstrated because neutrophils were not determined).

*PI3, IL10, MSR1*, and *CD86* were the gene biomarkers that best discriminated controls from NA patients with severe clinical features. *IL10* has pleiotropic effects in immunoregulation and inflammation (33). It has been extensively related with asthma and allergy diseases (34).

*MSR*1 or macrophage scavenger receptor type I, or CD204 has been described in many cell locations (usually in tissues), such as vascular smooth muscle cells, endothelial cells, human lung epithelial cells, etc. (35). This fact increases its pathophysiological potential, and has been described as a central pivot of health and disease (36). MSR1 was associated with asthma and was postulated by our group as a very good biomarker candidate for severity in several respiratory diseases (21).

*CD86* or *B7.2*, encodes a type I membrane protein expressed on antigen-presenting cells (APCs) and which provides costimulatory signals necessary for the initiation, modulation, and regulation of an effective T cell response. Most APCs constitutively express low levels of CD86, but following activation they are rapidly upregulated (37). Interestingly, in this study, *CD86* was the only gene biomarker capable of discriminating severe NA from moderate–mild NA patients, featuring a good AUC value (0.76). *CD86* was followed by *MSR1* (AUC: 0.66), *CPA3* (0.65), and *PHLDA1* (0.62), which were classified as regular biomarkers for this discrimination. *CPA3* or carboxypeptidase A3, is a metalloexopeptidase specifically expressed by mast cells (38). *CPA3* was described as the best individual discriminator for eosinophilic asthma in a study of six gene biomarkers in sputum (39). *PHLDA1* is a nuclear protein that has been postulated as a biomarker in the early detection and/or therapy of gastric cancer (40), but never before has been associated with asthma disease. Overall, these gene analyses strengthen our previous results and evidence the potential of these nine gene biomarkers. For that, the next step was to determine the effectiveness of these biomarkers at the protein level using when it was available (IL-10, CHI3L1, IL-8, and PI3) the serum-ELISA as a quantitative assay that is commonly useful to analyze soluble biomarkers due to its sensitivity, specificity, and simplicity. On the other hand, although our gene expression results did not revealed *POSTN* as a differential gene, we decided to include the analysis of periostin levels in the serum, as it is one of the main biomarkers described as indicator of Th2-inflammation (41, 42) and the serum periostin levels have been related to the response to anti-IL-13 therapy in patients with moderate–mild asthma (43).

The individual protein biomarkers results are summarized in Table 2. MSR1’s lower band and SERPINB2 were the best individual biomarkers for discriminating the NA group and its severity from healthy control subjects. *SERPINB2* is a member of the group of inhibitors of the serine protease family, enzymes that inhibit protease cathepsin G neutrophils and chymase of mast cells. *SERPINB2* has been detected in different cell types, playing a role in inflammation and remodeling (44). It has been tentatively suggested that SERPINB2 represents a novel effector of the multiple airway remodeling actions provoked by IL-13 (45). It has been described, together with *POSTN* and chloride channel accessory 1, as a gene-signature for Th2 asthma and mainly IL-13 asthma phenotype (41), but until our knowledge, SERPINB2 protein expression has only been studied in broncoalveolar lavage (BAL) and never before at a peripheral level.

IL-8 was the only individual protein biomarker with a good predictive accuracy for discriminating clinical severity between moderate–mild vs severe patients (AUC: 0.76), although with a moderate statistical power (CI 95%, 0.49–1). These results could highlight the relevance of IL-8 and indirectly confirm the recent publication in BAL, describing that neutrophils and IL-8 are the only inflammatory components that distinguish controlled from uncontrolled asthma (46), but should be confirmed in a larger population. In this regard, novel small molecules targeting neutrophilic inflammation, such as chemokine (CXC) receptor 2 (CXCR2) antagonists have been analyzed in the noneosinophilic asthma context, showing how these antagonists reduce neutrophils, but do not improve clinical outcomes in studies to date (47). Ligands for the CXCR2 receptor include the chemokines CXCL8 (IL-8). Recent studies indicate that while selective CXCR2 antagonists were found to significantly lower airway neutrophil counts in a mechanistic 1-month pilot study in more severe asthmatics (48), a lack of efficacy was observed in a larger 6-month clinical Ph2 trial [*n* = 640] specifically targeting CXCR2/IL-8 pathway in this defined asthma population (49).

The next step was to analyze the change in the predictive results after combining two (Table 3A–D) or three biomarkers (Table 4A and B). Table 5 shows the global analyses, with the ranking of the best individual or combined biomarkers for each of the comparisons performed. Overall, we had very good AUC results, and here we propose four protein panels of individual biomarkers as well as different combinations of biomarkers for the diagnosis and/or prognosis of NA using peripheral samples and standard techniques. We would like to highlight the combination of CHI3L1 + IL-8 + POSTN for discriminating moderate–mild NA from severe NA (Table 5 and Table 4B), as this had the best AUC (0.98). Previously, serum concentrations of CHI3L1 were associated with the severity of asthma and were inversely correlated with lung function and FEV_1_ (50), indicating that serum CHI3L1 was important in the specific inflammatory phenotype of asthma. However, more recently it has been described in a large group of patients with asthma, that serum concentrations of CHI3L1 were only slightly increased in those with the most severe asthma (51). Interestingly, contrasting with those previous results, in this study CHI3L1 protein levels were higher in moderate–mild patients compared with severe asthmatic patients (Figure 1), and, together with the levels of IL-8 and periostin, could be tested in an easy and reproducible ELISA to verify predictable disease severity in nonallergic patients. Although these results are very encouraging, we believe they should be tested in larger populations, with the same age-range and by different groups in order to test their reproducibility and to validate them.

In summary, in this work we have tried to define the relevance at gene and protein level of a set of biomarkers in peripheral samples. Our ultimate objective is to provide new and useful diagnostic and therapeutic tools for NA.

## Author Contributions

SB, DC, and BC have worked in all project-steps: design of study, experimental work, results discussion, and manuscript elaboration. LC-J collaborated in the manuscript elaboration. JS, CP, JQ, and FF performed the patient’s selection and collaborated in the design of study. CL has collaborated in design of study, results discussion, and manuscript elaboration.

## Conflict of Interest Statement

The authors declare that they have no conflicts of interest. The data published in this report are being evaluated for protection (Request Number: P201730947).

## References

[1] GINA. Global Initiative for Asthma. Global Strategy for Asthma Management and Prevention. (2017). Available from: http://www.ginasthma.org/

[2] Mukherjee AB Zhang Z Allergic asthma: influence of genetic and environmental factor. J Biol Chem (2011) 286:32883–9. 10.1074/jbc.R110.197046 21799018 PMC3190897

[3] Collins FS Varmus H. A new initiative on precision medicine. N Engl J Med (2015) 372:793–5. 10.1056/NEJMp1500523 25635347 PMC5101938

[4] D’Amato G Stanziola A Sanduzzi A Liccardi G Salzillo A Vitale C Treating severe allergic asthma with anti-IgE monoclonal antibody (omalizumab): a review. Multidiscip Respir Med (2014) 9:23. 10.1186/2049-6958-9-23 24735949 PMC4113133

[5] Holgate ST Stratified approaches to the treatment of asthma. Br J Clin Pharmacol (2012) 76:277–91. 10.1111/bcp.12036 PMC3731602 23163316

[6] Peters SP. Asthma phenotypes: nonallergic (intrinsic) asthma. J Allergy Clin Immunol Pract (2014) 2:650–2. 10.1016/j.jaip.2014.09.006 25439352

[7] Novak N Bieber T Allergic and nonallergic forms of atopic diseases. J Allergy Clin Immunol (2003) 112:252–62. 10.1067/mai.2003.1595 12897728

[8] Green BJ Wiriyachaiporn S Grainge C Rogers GB Kehagia V Lau L Potentially pathogenic airway bacteria and neutrophilic inflammation in treatment resistant severe asthma. PLoS One (2014) 9(6):e100645. 10.1371/journal.pone.0100645 24955983 PMC4067344

[9] Uddin M Nong G Ward J Seumois G Prince LR Wilson SJ Prosurvival activity for airway neutrophils in severe asthma. Thorax (2010) 65:684–9. 10.1136/thx.2009.120741 20685741

[10] Peters MC Mekonnen ZK Yuan S Bhakta NR Woodruff PG Fahy JV. Measures of gene expression in sputum cells can identify TH2-high and TH2-low subtypes of asthma. J Allergy Clin Immunol (2014) 133:388–94. 10.1016/j.jaci.2013.07.036 24075231 PMC3981552

[11] Green RH Brightling CE Woltmann G Parker D Wardlaw AJ Pavord ID. Analysis of induced sputum in adults with asthma: identification of subgroup with isolated sputum neutrophilia and poor response to inhaled corticosteroids. Thorax (2002) 57:875–9. 10.1136/thorax.57.10.875 12324674 PMC1746199

[12] Bullens DM Truyen E Coteur L Dilissen E Hellings PW Dupont LJ IL-17 mRNA in sputum of asthmatic patients: linking T cell driven inflammation and granulocytic influx? Respir Res (2006) 7:135. 10.1186/1465-9921-7-135 17083726 PMC1636037

[13] Simpson JL Gibson PG Yang IA Upham J James A Reynolds PN Impaired macrophage phagocytosis in non-eosinophilic asthma. Clin Exp Allergy (2013) 43:29–35. 10.1111/j.1365-2222.2012.04075.x 23278878

[14] Raedler D Ballenberger N Klucker E Böck A Otto R Prazeres da Costa O Identification of novel immune phenotypes for allergic and nonallergic childhood asthma. J Allergy Clin Immunol (2015) 135:81–91. 10.1016/j.jaci.2014.07.046 25226851

[15] Boyman O Kaegi C Akdis M Bavbek S Bossios A Chatzipetrou A EAACI IG biologicals task force paper on the use of biologic agents in allergic disorders. Allergy (2015) 70:727–54. 10.1111/all.12616 25819018

[16] Radonjic-Hoesli S Valent P Klion AD Wechsler ME Simon HU. Novel targeted therapies for eosinophil-associated diseases and allergy. Annu Rev Pharmacol Toxicol (2015) 55:633–56. 10.1146/annurev-pharmtox-010814-124407 25340931 PMC4924608

[17] Singh D Ravi A Southworth T CRTH2 antagonists in asthma: current perspectives. Clin Pharmacol (2017) 15:165–73. 10.2147/CPAA.S119295 PMC5733922 29276415

[18] Bateman ED O’Brien C Rugman P Luke S Ivanov S Uddin M Efficacy and safety of the CRTh2 antagonist AZD1981 as add-on therapy to inhaled corticosteroids and long-acting β_2_-agonists in patients with atopic asthma. Drug Des Devel Ther (2018) 4:1093–106. 10.2147/DDDT.S147389 PMC5942163 29765200

[19] Bateman ED Guerreros AG Brockhaus F Holzhauer B Pethe A Kay RA Fevipiprant, an oral prostaglandin DP_2_ receptor (CRTh2) antagonist, in allergic asthma uncontrolled on low-dose inhaled corticosteroids. Eur Respir J (2017) 50(2). 10.1183/13993003.00670-2017 28838980

[20] Muraro A Lemanske RF Hellings PW Akdis CA Bieber T Casale TB Precision medicine in patients with allergic diseases: airway diseases and atopic dermatitis—PRACTALL document of the European Academy of Allergy and Clinical Immunology and the American Academy of Allergy, Asthma & Immunology. J Allergy Clin Immunol (2016) 137:1347–58. 10.1016/j.jaci.2016.03.010 27155030

[21] Baos S Calzada D Cremades L Sastre J Quiralte J Florido F Data set on a study of gene expression in peripheral samples to identify biomarkers of severity of allergic and nonallergic asthma. Data Brief (2016) 10:505–10. 10.1016/j.dib.2016.12.035 28054016 PMC5196092

[22] Baos S Calzada D Cremades L Sastre J Quiralte J Florido F Biomarkers associated with disease severity in allergic and nonallergic asthma. Mol Immunol (2017) 82:34–45. 10.1016/j.molimm.2016.12.012 28011367

[23] GEMA (Guía española del manejo del asma). Arch Bronconeumol (2009) 45:2–35. 10.1016/S0300-2896(09)73459-3

[24] Jia G Erickson RW Choy DF Mosesova S Wu LC Solberg OD Periostin a systemic biomarker of eosinophilic airway inflammation in asthmatic patients. J Allergy Clin Immunol (2012) 130:647–54. 10.1016/j.jaci.2012.06.025 22857879 PMC3626285

[25] Parulekar AD Atik MA Hanania NA. Periostin, a novel biomarker of Th2-driven asthma. Curr Opin Pulm Med (2014) 20:60–5. 10.1097/MCP.0000000000000005 24247042

[26] Gao J Garulacan LA Storm SM Opiteck GJ Dubaquie Y Hefta SA Biomarker discovery in biological fluids. Methods (2005) 35:291–302. 10.1016/j.ymeth.2004.08.020 15722225

[27] Tsai YS Tseng YT Chen PS Lin MC Wu CC Huang MS Protective effects of elafin against adult asthma. Allergy Asthma Proc (2016) 37:15–24. 10.2500/aap.2016.37.3932 26932165

[28] Verrier T Solhonne B Sallenave JM García-Verdugo I. The WAP protein Trappin-2/Elafin: a handyman in the regulation of inflammatory and immune responses. Int J Biochem Cell Biol (2012) 44:1377–80. 10.1016/j.biocel.2012.05.007 22634606

[29] Lee CG Da Silva CA De la Cruz CS Ahangari F Ma B Kang MJ Role of chitin and chitinase/chitinase-like proteins in inflammation, tissue remodeling, and injury. Annu Rev Physiol (2011) 73:479–501. 10.1146/annurev-physiol-012110-142250 21054166 PMC3864643

[30] Prakash M Bodas M Prakash D Nawani N Khetmalas M Mandal A Diverse pathological implications of YKL-40: answers may lie in ‘outside-in’ signaling. Cell Signal (2013) 25:1567–73. 10.1016/j.cellsig.2013.03.016 23562456

[31] Hinks TS Brown T Lau LC Rupani H Barber C Elliott S Multidimensional endotyping in patients with severe asthma reveals inflammatory heterogeneity in matrix metalloproteinases and chitinase 3-like protein 1. J Allergy Clin Immunol (2016) 138:61–75. 10.1016/j.jaci.2015.11.020 26851968 PMC4929135

[32] Allen TC Kurdowska A. Interleukin 8 and acute lung injury. Arch Pathol Lab Med (2014) 138:266–9. 10.5858/arpa.2013-0182-RA 23782136

[33] Mingomataj EC Bakiri AH. Regulator versus effector paradigm: interleukin-10 as indicator of the switching response. Clin Rev Allergy Immunol (2016) 50:97–113. 10.1007/s12016-015-8514-7 26450621

[34] Palomares O Martín-Fontecha M Lauener R Traidl-Hoffmann C Cavkaytar O Akdis M Regulatory T cells and immune regulation of allergic diseases: roles of IL-10 and TGF-β. Genes Immun (2014) 15:511–20. 10.1038/gene.2014.45 25056447

[35] Tomokiyo R Jinnouchi K Honda M Wada Y Hanada N Hiraoka T Production, characterization, and interspecies reactivities of monoclonal antibodies against human class A macrophage scavenger receptors. Atherosclerosis (2002) 161:123–32. 10.1016/S0021-9150(01)00624-4 11882324

[36] Kelley JL Ozment TR Li C Schweitzer JB Williams DL. Scavenger receptor-A (CD204): a two-edged sword in health and disease. Crit Rev Immunol (2014) 34:241–61. 10.1615/CritRevImmunol.2014010267 24941076 PMC4191651

[37] Sharpe AH Freeman GJ The B7-CD28 superfamily. Nat Rev Immunol (2002) 2:116–26. 10.1038/nri727 11910893

[38] Trivedi NN Caughey GH Mast cell peptidase: chameleons of innate immunity and host defense. Am J Respir Cell Mol Biol (2010) 42:257–67. 10.1165/rcmb.2009-0324RT 19933375 PMC2830402

[39] Baines KJ Simpson JL Wood LG Scott RJ Fibbens NL Powell H Sputum gene expression signature of 6 biomarkers discriminates asthma inflammatory phenotypes. J Allergy Clin Immunol (2014) 133:997–1007. 10.1016/j.jaci.2013.12.1091 24582314

[40] Zhao P Lu Y Liu L. Correlation of decreased expression of PHLDA1 protein with malignant phenotype of gastric adenocarcinoma. Int J Clin Exp Pathol (2015) 8:5230–5. 26191222 PMC4503094

[41] Woodruff PG Modrek B Choy DF Jia G Abbas AR Ellwanger A T-helper type 2-driven inflammation defines major subphenotypes of asthma. Am J Respir Crit Care Med (2009) 180:388–95. 10.1164/rccm.200903-0392OC 19483109 PMC2742757

[42] Woodruff PG Boushey HA Dolganov GM Barker CS Yang YH Donnelly S Genome-wide profiling identifies epithelial cell genes associated with asthma and with treatment response to corticosteroids. Proc Natl Acad Sci U S A (2007) 104:15858–63. 10.1073/pnas.0707413104 17898169 PMC2000427

[43] Corren J Lemanske RF Hanania NA Korenblat PE Parsey MV Arron JR Lebrikizumab treatment in adults with asthma. N Engl J Med (2011) 365:1088–98. 10.1056/NEJMoa1106469 21812663

[44] Swartz JM Byström J Dyer KD Nitto T Wynn TA Rosenberg HF. Plasminogen activator inhibitor-2 (PAI-2) in eosinophilic leukocytes. J Leukoc Biol (2004) 76:812–9. 10.1189/jlb.0304182 15277569

[45] Cohn L Elias JA Chupp GL. Asthma: mechanisms of disease persistence and progression. Annu Rev Immunol (2004) 22:789–815. 10.1146/annurev.immunol.22.012703.104716 15032597

[46] Hosoki K Ying S Corrigan C Qi H Kurosky A Jennings K Analysis of a panel of 48 cytokines in BAL fluids specifically identifies IL-8 levels as the only cytokine that distinguishes controlled asthma from uncontrolled asthma, and correlates inversely with FEV_1_. PLoS One (2015) 10(5):e0126035. 10.1371/journal.pone.0126035 26011707 PMC4444276

[47] Thomson NC. Novel approaches to the management of noneosinophilic asthma. Ther Adv Respir Dis (2016) 10:211–34. 10.1177/1753465816632638 26929306 PMC5933607

[48] Watz H Uddin M Pedersen F Kirsten A Goldmann T Stellmacher F Effects of the CXCR2 antagonist AZD5069 on lung neutrophil recruitment in asthma. Pulm Pharmacol Ther (2017) 45:121–3. 10.1016/j.pupt.2017.05.012 28549850

[49] O’Byrne PM Metev H Puu M Richter K Keen C Uddin M Efficacy and safety of a CXCR2 antagonist, AZD5069, in patients with uncontrolled persistent asthma: a randomised, double-blind, placebo-controlled trial. Lancet Respir Med (2016) 4:797–806. 10.1016/S2213-2600(16)30227-2 27574788

[50] Chupp GL Lee CG Jarjour N Shim YM Holm CT He S A chitinase-like protein in the lung and circulation of patients with severe asthma. N Engl J Med (2007) 357:2016–27. 10.1056/NEJMoa073600 18003958

[51] Hansen JW Thomsen SF Porsbjerg C Rasmussen LM Harmsen L Johansen JS YKL-40 and genetic status of CHI3L1 in a large group of asthmatics. Eur Clin Resp J (2015) 2:25117. 10.3402/ecrj.v2.25117 26672955 PMC4653313

